# Randomized, controlled, parallel-group prospective study to investigate the clinical effectiveness of early insulin treatment in patients with latent autoimmune diabetes in adults

**DOI:** 10.1186/1472-6823-8-8

**Published:** 2008-07-24

**Authors:** Sinead Brophy, Helen Davies, Stephen Bain, Jeffrey W Stephens, Wei-yee Cheung, Kez Richards, Kathie Wareham, Charles Beaverstock, Janet Lloyd, Don Page, Meurig Williams, Ian Russell, Rhys Williams

**Affiliations:** 1 School of Medicine, Swansea University, Swansea, Wales, UK; 2 Clinical Research Unit, Swansea NHS Trust. Swansea, Wales, UK; 3 Diabetes Unit, Neath Port Talbot Hospital, Neath Port Talbot, Wales, UK; 4 Diabetes UK Cymru, Argyle House Castlebridge, Cowbridge, Cardiff, CF11 9AB, UK; 5 Diabetes Centre, Prince Philip Hospital, Llanelli, Carmarthenshire, Wales, UK; 6 Institute for Medical and Social Care Research, University of Wales, Bangor, Wales, UK

## Abstract

**Background:**

Latent autoimmune diabetes in adults [LADA] is a type 1 diabetes that is slowly developing. This means many people are treated as having type 2 diabetes at diagnosis as they are adults who are not immediately insulin dependent. LADA can be distinguished from type 2 diabetes by antibody tests. Patients who are antibody positive have an autoimmune reaction which is similar to that of type 1 diabetes and is not found in type 2 diabetes. We would like to examine the best way of treating LADA in the early phase of the conditions, with tablets (similar to type 2 diabetes) or with insulin (similar to type 1 diabetes).

**Methods/design:**

This is an open parallel group prospective randomised trial. Participants need to have a GAD antibody test results of 101 WHO units or more and a diagnosis of diabetes not requiring insulin at diagnosis. Participants will need to have been diagnosed within 12 months and not treated with insulin at study entry. They will be randomised to receive either insulin (NovoMix 30) or tablets (diet treated followed by metformin followed by glitazone (with or without metformin) followed by insulin). Primary outcome assessment will be for change in HbA1c and change in fasting C-peptide over 24 months. Secondary outcome measures will include Quality of life, GAD antibody levels, adverse events, inflammatory markers, insulin resistance, and markers of the metabolic syndrome.

**Discussion:**

This study seeks the best treatment for early LADA in terms of maintaining glycaemic control and maintaining natural insulin production.

**Trial registration:**

ISRCTN63815121

## Background

### Type 1 diabetes

Diabetes mellitus is the most common endocrine disease. There are two main types of diabetes, type 1 and type 2. Although type 1 diabetes often develops in young people, it can occur at any age. Type 1 diabetes is caused by an autoimmune process resulting in a selective destruction of the pancreatic insulin-secreting β-cells. The destruction of β-cells leads to hyperglycaemia due to a lack of insulin. The immune changes are reflected by the presence of islet cell antibodies (ICA) and antibodies to glutamic acid decarboxylase (GAD) [1]

### Type 2 diabetes

Type 2 diabetes most often develops in adults and is characterized by insulin resistance and subsequent insulin deficiency. Approximately 10% of patients with type 2 diabetes have antibodies to GAD and/or ICA and progress to insulin dependency [2,3]. The term LADA (latent autoimmune diabetes in adults) has been introduced to define adult diabetes patients who are initially non-insulin-requiring but with immune markers of type 1 diabetes, who progress to insulin dependency.

#### LADA

Latent autoimmune diabetes in adults is a type 1 diabetes which shows slow progression to insulin dependence instead of acute onset as is seen in 'classical' type 1 diabetes. It is an autoimmune condition unlike type 2 diabetes and therefore can be distinguished from type 2 by blood tests for antibodies.

#### Treatment

Early studies in Japan (n = 58) [4-6] have suggested that LADA patients should start insulin within 1 year of diagnosis in order to maintain near normoglycemic control. It is also suggested that this treatment can prevent slowly progressive beta-cell failure. Patients taking insulin had an improved C-peptide response (i.e. improved Beta cell function and natural insulin production), stable HbA1_c _values and reduced auto-antibody level.

However, oral agents such as glitazones have anti-inflammatory activity and could potentially also be an effective treatment for patients in the non-insulin dependent stage of their diabetes. Rosiglitazone given in combination with insulin was found to maintain C-peptide levels better than insulin alone [7]. Laboratory studies support an anti-inflammatory role for glitazones which could be beneficial for people with LADA [8].

Sulphonylurea treatment may be harmful to people with LADA as they may deplete the already low reserves of insulin. In some studies, LADA patients given sulphonylurea (with or without insulin) have persistent antibodies and poor fasting glucose concentrations compared to people on insulin alone [5,9,10]

The team at the School of Medicine in Swansea University [SB, HD and RW] have undertaken a Cochrane systematic review to examine interventions for LADA [11] which found that sulphonylurea should not be used in LADA but there is no significant evidence for or against other lines of treatment of LADA. To date there are no other systematic reviews.

## Methods/design

### Aims and objectives

To compare the effectiveness of treatment regimes for type 1 diabetes compared to regimes for type 2 diabetes in LADA.

#### Primary objectives

To examine the effect of standard treatment for type 1 (insulin [NovoMix 30] therapy) compared with standard treatment for type 2 diabetes (tablets), on (1) change in fasting serum C-peptide level over 24 months and (2) change in HbA1c level over 24 months in patients with LADA.

#### Secondary objectives

To assess the effect of standard type 1 treatment (insulin) on average number of times the fasting plasma glucose level is above 8 mmol/l (141 mg/dl), quality of life [ADDQol [12]], proportion of patients with insulin dependence (as judged by C peptide level below 0.38 ng/ml [13]), GAD antibody level, HOMA, hypoglycaemic events (particularly hypoglycaemic events), weight/blood pressure/total cholesterol and inflammatory markers in LADA patients compared to standard type 2 diabetes treatment.

### Design

This trial is a multi-centre open parallel group prospective randomised trial. Participants are recruited from their primary health care practitioner following a routine test for GAD antibodies. They are seen in secondary care at 3 centres in Wales (Swansea, Carmarthenshire and Cardiff). The protocol has been approved by the Multi-Centre Research Ethics Committee for Wales. Each recruited subject has to sign a consent form and give their consent to randomisation.

#### Intervention

**Figure 1:**
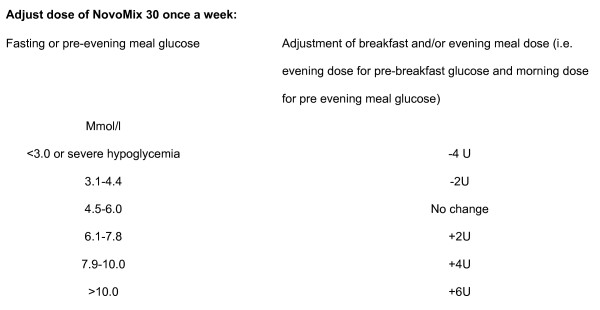
Insulin treatment.

Insulin arm: Patients will be given advice on diet and exercise and life style and will be started on NovoMix 30, one dose of 6 U at the evening/main meal. Dose will be adjusted in increments of 2–6 U depending on fasting glucose level [See Figure 1]. When total dose equals 16 U patient will be started on 4 units with breakfast and continue with 16 units with evening meal. Breakfast and/or evening meal dose will be adjusted where necessary at increments of 2–6 U depending on fasting and/or pre-evening meal glucose level. [See Figure 1 below]. Patient needs to keep a daily diary of insulin doses taken.

All patients will see a dietician before insulin treatment is initiated. Patients will be contacted weekly by phone in the first month, fortnightly in the second month and once a month thereafter. Patients will monitor their blood glucose twice a day for 3 days of the week.

#### Tablet arm:

##### [Figure 2] Step 1

**Figure 2:**
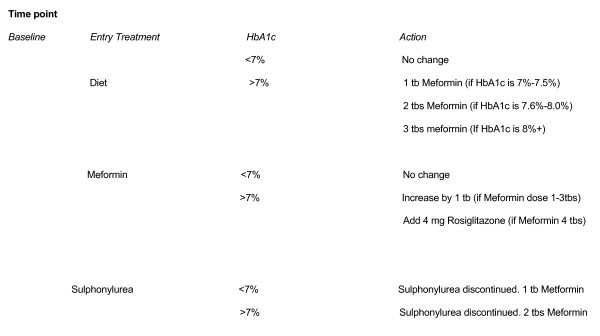
Standard treatment arm.

Patient's not treated with tablets will be given a 3 months trial of lifestyle modification including advice on diet, exercise and smoking. If HbA1_c _remains at 7% or above the patient will progress to step 2. Patients will be progressed to step 2 if their GP has already put them on metformin (put in at number of metformin tables that GP has recommended) or sulphonylurea or if their baseline HbA1_c _is above 8% (patients will start on one 500 mg tablet per day). This means that sulphonylurea will be discontinued in all patients. Patients on step 1 will be progressed to step 2 before the 3 month period if they have already had a 3 month period of diet before starting the study AND during phone contact they report to be symptomatic (thirst, fatigue, polyuria) and unwell. In these cases, patients may be invited to attend clinic before the 3 month period to be given metformin.

##### Step 2

Life style modification and Metformin. If the patient has a HbA1_c _of 7%–7.5% they start on 500 mg × 1 per day for the next 3 months. If the HbA1_c _is 7.6%–8.0% they would start the first week on 500 mg × 1 day and then for the remaining period of the three months will take 500 mg × 2 day. If the HbA1_c _is above 8.0% then patient will be on 500 mg × 3 per day. This will be titrated at the rate of 500 mg × 1 for the first week, 500 mg × 2 for the second week and 500 mg × 3 for the remaining time of the 3 month period. If unable to tolerate metformin then the participant will be given Glucophage SR and if unable to tollerate this they will then progress to Step 3. If HbA1_c _remains at 7% or above then those on 1 tablet per day will be moved to 2 tablets per day, those on 2 tablets per day will be moved to 3 tablets per day, those on 3 tablets per day will be move to maximum dose of 2 gms per day. If after 3 months the HbA1_c _is above 7% even with maximum dose, then progress to Step 3. If during telephone contact the patient report symptoms then they may be titrated up. In summary, patients will be have their HbA1c evaluated every 3 months and will be titriated accordingly.

##### Step 3

Life style modification and Glitazone [Rosiglitazone]. Patients with a HbA1_c _of 7% or above will be given 4 mg once per day for 3 months. If after a 3 month period the HbA1_c _remains at 7% or above then titrate to maximal dose of 4 mg twice per day with or without Metformin. If HbA1_c_remains at 7% or above for an additional 3 months then move to step 4. Therefore, glitazone monotherapy will be used if Metformin intolerant otherwise glitazone will be added to the metformin (as per standard practice). Before initiation of Rosiglitazone a repeat medical history will be taken with special emphasis on cardiovascular disease, if patients have a history of cardiovascular disease (defined as IHD, PVD) they will not be initiated on Rosiglitazone but will move directly to Step 4 (insulin). Patients on rosiglitazone will be monitored using the adverse events form, for symptoms of heart disease specifically for chest pain and shortness of breath. Any adverse events suggestive of heart disease will result in Rosiglitazone being discontinued and the patient moving to step 4.

##### Step 4

Life style modification and insulin therapy (oral agents will be stopped). Titration will be based on fasting blood glucose level (as given in Figure 1). The initiation of insulin will the same as for the insulin arm and will follow the protocol detailed above.

All tablet treated patients will be asked to keep a diary of daily medication and record blood glucose levels weekly.

All changes in medication will be notified to the patients GP.

### Study population and inclusion/exclusion criteria

Subject are recruited from their primary care practitioner if they have a diagnosis of diabetes with a GAD antibody result of 101 WHO units or more.

#### Inclusion criteria

1. Male, non-fertile female (i.e., post menopausal, post hysterectomy, or sterilized by tubal ligation) or female of childbearing potential using a medically approved birth control method.

2. The patient has a diagnosis of diabetes mellitus according to WHO classification.

3. The patient has a positive GAD antibody test of 101 WHO units or more on two separate occasions.

4. Age 18 +

5. The patient did not start on insulin within 1 month of diagnosis

6. Written informed consent to participate in the study.

7. Ability to comply with all study requirements.

#### Exclusion criteria

1. Pregnant or breast-feeding females and females who plan pregnancy or breast-feeding during the course of the study.

2. A history of:

▪ Diabetes that is a result of pancreatic injury, or secondary forms of diabetes, e.g., Cushing's syndrome and acromegaly.

▪ Acute metabolic diabetic complications such as ketoacidosis or hyperosmolar state (coma) within the past 6 months

3. Acute infections, which may affect blood glucose control within 4 weeks prior to visit 1.

4. Malignancy including leukaemia and lymphoma (not including basal cell skin cancer) within the last 5 years.

5. The patient has a known immune deficiency from any disease, or a condition associated with an immune deficiency.

6. The patient is receiving immunosuppressive or immunomodulating agents or cytotoxic therapy, or any medication that, in the opinion of the site investigator, might interfere with the study.

7. Any of the following significant laboratory abnormalities:

• Patients with severe renal failure as defined previous renal transplant or currently having renal dialysis or GFR < 30.

• Clinically significant laboratory abnormalities, confirmed by repeat measurement, that may interfere with the assessment of safety and/or efficacy of the study drug, other than hyperglycemia and glycosuria at visit 1.

• Severe ketonuria (+++ on urine sticks testing; ++ on repeated urine sticks testing).

8. The patient is a known or suspected drug abuser.

9. The patient has chronic hepatitis or liver cirrhosis, or any other chronic liver disease.

10. The patient is known to test positive for hepatitis B antigens or hepatitis C antibodies

11. The patient is known to test positive for HIV antibodies.

12. The patient has any significant diseases or conditions, including psychiatric disorders and substance abuse that, in the opinion of the site investigator, are likely to affect the patient's response to treatment or their ability to complete the study.

13. The patient has chronic haematological disease.

14. The patient has had a severe blood loss (≥ 400 mL, e.g., blood donation) within 2 months before the first dosing of the study medication.

15. The patient has known proliferative retinopathy.

16. Patient has had stage 3–4 heart failure.

17. The patient is participating in another research study which may affect the results of this trial.

### Withdrawn from study

A patient is considered to have withdrawn from the study if they completely withdraw from the study, i.e.:

- Move and do not provide a new address or contact number

- Miss more than 3 visits without contacting the study centre.

- Die

However, if they discontinue treatment but continue participate in data collection they will not be considered to have withdrawn, i.e:

- Want to change therapy due to personal reasons but attend a termination visit.

- Experience a serious adverse event that in the medical judgement of the site investigator and for the best interest of the subject, are grounds for discontinuation, but attend a termination visit.

- Withdraw consent but give consent to the use of the data collected to date.

- Develop a concomitant condition which is given in the exclusion criteria but do attend a termination visit.

If a patient discontinues the study prematurely at any time after entering the study, attempts should be made to conduct a termination final visit at which time all of the assessments listed for the final visit will be performed.

The reason for premature discontinuation and – if applicable – the final visit will be documented.

### Follow up procedure

**Figure 3:**
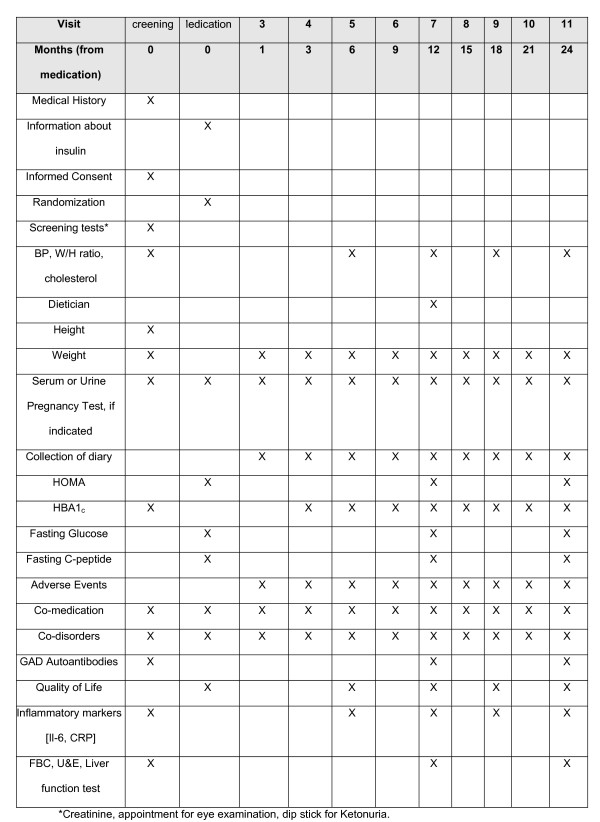
Schedule of visits.

Patients are seen for a screening visit where they are asked to give consent and have baseline tests regarding inclusion/exclusion criteria. They return after 2 weeks for randomisation and are followed every 3 months there after. [See Figure 3]

### Analysis plan

The statistical evaluation will be performed using SPSS. All data will be listed and trial summary tables will be provided. Summary statistics will be presented by group.

The primary analysis will be an intention to treat analysis examining (1) the average change in C peptide level at the end of the study in both groups and (2) the average change in HbA1c level at the end of the study in both groups. A linear regression model will be used to examine the effect of baseline GAD level, age, sex and baseline fasting C-peptide or HbA1c level as covariate and a treatment term on the change in HbA1c and C-peptide levels. In addition, repeated measures analysis (area under the curve, maximum minus minimum, time taken to maximum, time taken to minimum and slope) for C-peptide and HbA1c will be performed. ANOVA for repeated measurements with be used when variables are normally distributed. Otherwise, log transformations or non parametric tests will be employed.

Data will be collected by research nurses under the medical care of Professor Stephen Bain and Dr Jeffrey Stephens and monitored by the trial co-ordinator. The analysis will be performed by Dr Wai-yee Cheung who will be blind to treatment group until after all analysis has been completed and interpreted. The CONSORT statement will be followed and used for reporting results.

Secondary analysis will include: Intention to treat analysis of :

(1) Average change in fasting C-peptide level in the insulin group compared to the control (best alternative care) group at 12 months.

(2) Average change in HbA1c level in the insulin group compared to the standard care group at 12 month. Data will be presented using confidence intervals only.

(4) Quality of life in the insulin vs standard care groups

(5) Number of events of fasting glucose being above 8 in the insulin vs standard care arm.

(6) Proportion of patients with a C-peptide below 0.38 ng/ml at baseline, 12 months and 24 months.

(7) Level of inflammatory markers in the insulin vs standard care arm

(8) GAD levels of insulin vs standard care

(9) Episodes of adverse events in the insulin vs standard care arms

(10) Weight gain in terms of BMI and waist/hip ratio in both groups

(11) HOMA or insulin resistance in both groups

We shall use two sided tests and confidence intervals for all comparisons.

### Handling of missing and incomplete data

Data may or may not be missing at random. We shall compare patients with complete and missing data in sex, age and metabolic control (HbA1c) to test whether data are missing at random.

If data are missing at random and a participant does not attend follow-up visits, then the last recorded measure will be used as the final value. In cases were laboratory data is missing we will attempt to obtain values from the participants laboratory records supplied to their GP (using their NHS number in the diagnostics laboratory) unless they have actively withdrawn their consent for us to view their GP records.

If data are missing in a non-random way we shall compare the 'worst' case (high HbA1c and low c-peptide) and the 'best' case (normal HbA1c and normal c-peptide) to estimate confidence intervals for the final analysis.

### Sample size

The study conducted in China [14] examined fasting C-peptide levels over 12 month, the average difference in C-peptide at 12 months was 0.18 nmol/l (stdev: 0.5 nmol/l) between the insulin vs the SU groups. To find a difference of 0.7% in average HbA1c (standard deviation = 1.5%) we would need 97 analysible people in the insulin and the standard care arms of the study (power 90%, significance = 5%). This would detect an average difference of 0.2 nmol/l in fasting C-peptide. As the power calculation is based on patients in China, we will re-calculate the sample size based on the standard deviation of the first 30 patients recruited in our study. We will examine the standard deviation of the HbA1c and the fasting C-peptide in all 30 patients at 3 months (regardless of treatment) in order to re-estimate the sample size assuming a difference of 0.7% in HbA1c and 0.2 nmol/l in fasting C-peptide.

### Randomisation procedure

WYC will provide SB with a randomisation sequence from a computer randomisation programme [15]. The clinical nurse (KR) will see the patients and register their details on the registration database, before seeking their random allocation from SB.

Randomisation procedure: The randomisation sequence will be determined by a computer randomisation programme [15]

## Discussion

Within this study we introduce routine GADA testing at the general practice level for all newly people with diabetes. We provide newsletters, an internet site  and a helpline for people diagnosed with LADA. This is the first study in Europe examining the best first line treatment at the primary care level for the early management of people with LADA.

## Abbreviations

LADA: Latent autoimmune diabetes in adults; GAD: Glutamic Acid Decarboxylase; ICA: islet cell antibodies; HOMA: Homeostasis Model Assessment.

## Competing interests

This work is funded by Novo Nordisk Ltd, a commercial company that make insulin. They are supplying the insulin for this trial. The protocol went through the Novo Nordisk Scientific Committee before funding was approved. However, Novo Nordisk will not have any contact with patients, will not have access to results, or be involved interpreting, writing up or publication of the final results. This is an investigator lead trial and the running of the trial is independent of the funding organisation (Novo Nordisk Ltd).

## Authors' contributions

All authors have contributed to the design of the protocol giving advice for their own research expertise. StB and JWS have advised on treatment regimes, JL and DP advised on the protocol from the perspective of the patient, WYC and IR designed the analysis and statistical sections, KR, KW and MW advised on the implementation and running of the trial in the local area and SB, HD, CB and RW wrote the early drafts of the protocol in which other authors amended. All authors have read and approved the final manuscript.

## Pre-publication history

The pre-publication history for this paper can be accessed here:


